# Assessing the suitability of automated registration and segmentation for dosimetry calculations in SIRT treatment planning

**DOI:** 10.1186/s40658-026-00858-4

**Published:** 2026-05-17

**Authors:** Félix Quinton, Romain Popoff, Fabrice Meriaudeau, Jean-Marc Vrigneaud, Olivier Chevallier, Julie Pellegrinelli, Jean-Louis Alberini, Benoit Presles

**Affiliations:** 1 Université Bourgogne Europe, CNRS, ICMUB UMR 6302, 9 Avenue Alain Savary, 21000 Dijon, France; 2 Service de Médecine Nucléaire, Centre Georges-François Leclerc, 1 Rue du Professeur Marion, 21000 Dijon, France; 3 Service de Radiologie et Imagerie Médicale Diagnostique et Therapeutique, Centre Hospitalier Universitaire, 2 Boulevard Maréchal de Lattre de Tassigny, 21000 Dijon, France

**Keywords:** Dosimetry, Deep learning, Hepatocellular carcinoma, Segmentation, Selective Internal Radiation Therapy, Registration

## Abstract

****Background**:**

Selective internal radiation therapy (SIRT) increasingly relies on accurate magnetic resonance imaging (MRI) to computed tomography (CT) registration and accurate liver and tumour segmentation, for effective pre-treatment planning. This study evaluates the impact of automatic registration and segmentation techniques on dosimetry calculations for SIRT treatment planning. It compares semi-automatic and automatic registration, as well as manual and automatic deep learning-based segmentation.

****Methods**:**

Pre-treatment data from 90 patients with hepatocellular carcinoma were analysed. The dataset consisted of contrast-enhanced T1-weighted MRI scans with manually delineated liver and tumour volumes of interest (VOIs), as well as single-photon emission computed tomography (SPECT)/CT scans with manually or semi-automatically delineated liver VOI. The clinical routine pipeline, which involves semi-automatic registration and manual/semi-automatic segmentation, was used as the baseline pipeline and compared to experimental pipelines that use intensity-based deformable automatic registration or deep learning-based automatic segmentation. Dosimetric accuracy was assessed via metrics such as the mean absorbed dose, the minimum dose received by 70% of the volume (D70), and inverted cumulative dose-volume histograms.

****Results**:**

Semi-automatic and automatic MRI to CT liver registration achieved comparable Dice scores of 92%. However, tumour registration varied significantly between registration methods yielding average Dice scores of 79%. Multimodal tumour segmentation approaches outperformed monomodal ones, achieving average Dice scores of 66.6 versus 62.5%. Using the baseline pipeline, the average tumour absorbed dose per patient was 115.6 Gy. Using the fully automatic approach, tumour absorbed doses differed from baseline values by an average of 6.6 Gy. Differences ranged from 0.3 Gy in the best case to 202.8 Gy when the automatic tumour segmentation markedly deviated from the manual delineation. Finally, it was found that a Dice score of at least 80% was required to avoid statistically significant differences in absorbed dose estimates between the clinical and automatic approaches.

****Conclusion**:**

Semi-automatic and automatic registration show equivalent performance, allowing for complete automation. Automatic segmentation demonstrates promising results, with approximately 40% of patients achieving tumour Dice scores above the 80% threshold. 30% of cases show intermediate performance (60–80% tumour Dice scores), while the remaining 30% are still challenging. Further refinement of segmentation methods is required to enhance dosimetric accuracy.

## Background

Liver cancer is a major global health problem due to its high incidence and mortality rates, with more than 800,000 new cases diagnosed worldwide in 2022 [1]. Hepatocellular carcinoma (HCC) is the most common type of primary liver cancer [1], representing approximately $80\%$ of primary liver cancers. Various therapeutic options are available depending on the cancer’s stage. Tumour resection, ablation, and/or liver transplantation are the main options for early-stage HCC, while targeted therapies such as transarterial chemoembolisation or transarterial radioembolisation, also known as selective internal radiation therapy (SIRT), and systemic therapies are used for intermediate and advanced HCC [2].

In recent years, the indications for SIRT have significantly increased for unresectable HCC, particularly with the adoption of personalised dosimetric approaches [3]. SIRT involves the injection of yttrium-90-labelled microspheres (^90^Y-ms), a $\beta$-radiation emitter, directly into the hepatic arterial branches through selective catheterisation. Once administered, these microspheres are predominantly carried by the blood flow that supplies the tumour, where they become trapped in the capillaries, delivering highly localised radiation to the cancerous cells. This technique enables the delivery of a high radiation dose directly to the tumour while minimising radiation exposure to the surrounding non-tumoural liver tissue and other nearby organs at risk.

Prior to treatment, an injection of macro-aggregates of human albumin radiolabelled with technetium-99 m ($^{99\text {m}}$Tc-MAA) is performed under the same conditions as the future treatment to simulate it. This surrogate is made up of biodegradable particles ranging in diameter from 10 to $150~\mu m$. Ninety percent of these particles have a diameter between 10 and $40~\mu m$, and $1-2\%$ measure less than $15~\mu m$. A single-photon emission computed tomography combined with computed tomography (SPECT/CT) and planar single-photon emission acquisitions are then performed to visualise the distribution of $^{99\text {m}}$Tc-MAA within the tumour, non-tumour liver, and extra-hepatic organs [4, 5], to plan the treatment and calculate the appropriate activity of ^90^Y-ms to deliver [6].

According to European recommendations for SIRT dosimetry, accurate absorbed dose estimation requires the delineation of at least two hepatic regions: the tumour and the perfused liver [3]. This requirement reflects the need to separately assess dose delivery to tumour tissue and surrounding healthy liver. In practice, however, tumour dosimetry is particularly sensitive to the accuracy of tumour volume delineation. While the liver and lungs are generally well visualised on the CT component of SPECT/CT, tumour boundaries are often poorly defined without contrast enhancement, making direct tumour segmentation on SPECT/CT unreliable.

Several approaches exist for tumour segmentation, including manual delineation, threshold-based methods, and automated techniques. However, Nodariet al. [7] demonstrated that the most accurate strategy is to perform tumour segmentation on contrast-enhanced diagnostic images, where lesion visibility is optimal. Once delineated, the tumour volume is transferred to the SPECT/CT space for absorbed dose calculation via image registration. In clinical workflows, this is commonly achieved through magnetic resonance imaging (MRI) to CT registration guided by liver segmentations.

However, these delineation and registration steps are time-consuming and require the expertise of a trained radiologist. As a result, there is a need for automation in clinical workflows. Many studies in the literature have explored automatic registration [8–10] or automatic liver and tumour segmentation [11, 12]. Most of these studies focus primarily on anatomical accuracy and do not evaluate the impact of their proposed methods on dosimetry.

Still, some studies have directly examined the impact of automation on absorbed dose estimation. For instance, Li et al. [13] assessed an atlas-based auto-segmentation method for liver delineation on MR images in resin ^90^Y-based SIRT. Using normalised deformable registration and multi-atlas matching, they reported Dice scores of 80 to $83\%$ and activity ratios close to unity, indicating that atlas-based segmentation can substitute manual contouring for dosimetric calculations after physician review.

Luu et al. [14] proposed an almost fully automatic method for liver-lung shunt fraction (LSF) quantification in SIRT, combining CNN-based liver and lung segmentation with non-rigid registration to align contrast-enhanced and non-contrast-enhanced CT images. Validated on 60 HCC patients, the method achieved a median LSF error of $0.14\%$, substantially lower than manual 2D delineation (1.7–$1.8\%$) and a previous CNN-based approach ($0.42\%$).

Similarly, Tang et al. [15] investigated segmentation-guided multimodal liver image registration for dosimetry estimation in SIRT. By combining convolutional neural network (CNN) based liver segmentation with landmark-based registration methods, they showed improvements in aligning CT and MRI scans, facilitating more accurate dose estimations.

Jafargholi Rangraz et al. [16] aimed to improve dosimetric accuracy in SIRT by developing a semi-automatic multimodal segmentation approach for the liver, arterial perfusion territories, and tumours. Their method used a joint region-growing technique with $^{99\text {m}}$Tc-MAA SPECT/CT and/or ^90^Y positron emission tomography (PET), fluorine-18 fluorodeoxyglucose (^18^F-FDG) PET/CT, and contrast-enhanced cone-beam computed tomography (CBCT) to refine manual segmentation and improve the alignment of anatomical and perfusion data. This approach demonstrated that semi-automatic multimodal segmentation could support accurate treatment planning in SIRT, reduce manual workload, and improve consistency in absorbed dose calculations.

Finally, Stella et al. [17] proposed an automatic healthy liver segmentation method for ^166^Ho radioembolisation based on dual-isotope (^166^Ho–$^{99\text {m}}$Tc) SPECT. By exploiting the $^{99\text {m}}$Tc signal from Kupffer cells, the method delineates healthy parenchyma without manual registration and enables direct dosimetry on ^166^Ho images. Across 66 procedures, mean healthy liver doses were $20 \pm 8$ Gy, comparable to manual results ($18 \pm 7$ Gy) with a mean difference of ± 6 Gy. The approach markedly reduced segmentation time while maintaining dosimetric accuracy, supporting its use in automated ^166^Ho dosimetry workflows.

To the best of our knowledge, no studies have yet examined the combined effects of automatic liver registration and segmentation on absorbed dose calculations. Therefore, the present study will focus on evaluating the impact of automating these steps on treatment planning. Specifically, the relationship between automatic tumour segmentation and absorbed dose in SIRT will be thoroughly explored.

Traditional image registration methods are either feature-based or intensity-based. The former use landmarks such as keypoints [18] or segmentations [15], while the latter align voxel intensities [19, 20]. Registration typically starts with global alignment (rigid or affine), followed by deformable refinement using methods such as B-splines or Demons [21, 22], often within a multi-resolution framework [23]. Deep learning now offers alternatives by predicting transformations [24], using encoder-decoders [25] or generative adversarial networks [26].

Deep learning has greatly improved performance in segmentation tasks. Early methods adapted classification CNNs such as AlexNet [27] and ResNet [28], for segmentation purposes. A major breakthrough came with the introduction of the U-Net [29] model, an encoder-decoder model designed for biomedical images. More recently, transformer-based models [30, 31] have demonstrated impressive performance in various vision tasks. Both CNN and transformer models have achieved state-of-the-art results in medical image segmentation, particularly for organs and tumours [32, 33].

The main contributions of the present study are as follows:

- Evaluation of automatic registration and segmentation: Comparison of clinical semi-automatic registration with automatic registration, and manual/semi-automatic segmentation with automatic segmentation.
- Study of monomodal versus multimodal segmentation: Comparison of monomodal segmentation (using MRI only) and multimodal (using both MRI and SPECT images) for automatic liver and tumour delineation.
- Correlation between registration and/or segmentation performance and dosimetry: Evaluating the correlation between the quality of registration and segmentation performance, and the impact on treatment planning absorbed dose calculations.

## Methods

The methodological framework of this study relies on four distinct processing steps, combining clinical routine tools and research-oriented automatic methods. In clinical practice, semi-automatic MRI-to-CT registration, as well as manual segmentation of the tumours and manual or semi-automatic segmentation of the livers, were performed using the CE-marked MIM SurePlan™ LiverY90 software (MIM Software Inc., Cleveland, OH, USA), in the version available at the time of the study [34]. The automatic image registration process was carried out using the open-source Elastix software developed at the Image Sciences Institute, University Medical Centre Utrecht, The Netherlands [35]. The automatic liver and tumour segmentation was performed using nnUNet [36]. These automatic research tools have been developed for experimental purposes and are not CE-certified. They are currently intended for use as decision support systems or for secondary verification in a research context.

### Dataset and data acquisition

The dataset used in this study comprises diagnostic contrast-enhanced T1-weighted MRI images and $^{99\text {m}}$Tc-MAA images from 90 patients eligible for SIRT treatment for HCC. All data were obtained from patient acquisitions conducted at the University Hospital of Dijon and at the Georges-François Leclerc Cancer Research Centre, both in Dijon, France, between 2012 and 2024.

For each patient, the following data were included:

- **Contrast-enhanced T1-weighted MRI (CE-MRI) and volumes of interest (VOIs):** Diagnostic MRI images used by radiologists for liver and tumour segmentation. These data include manual delineations of the entire liver and tumours.
- $^{99} {\textbf {m}}$**Tc-MAA SPECT/CT and VOI**: SPECT/CT images used for treatment simulation, accompanied by manual/semi-automatic delineation of the liver. No tumour delineation is available because CT images were acquired without contrast agent.
- **Registration file:** A file containing the transformation data corresponding to the semi-automatic liver registration from MRI to CT. It allows to transfer the tumour VOI from the MRI to the CT by aligning the livers.

**Fig. 1:**
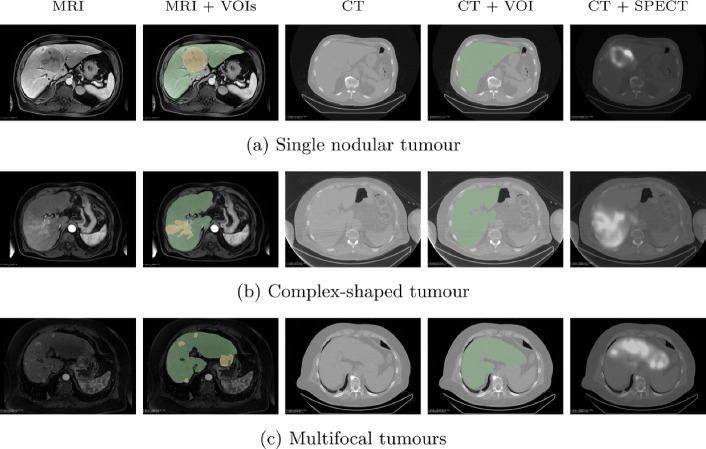
Axial slices from three patients of the ATLAS dataset illustrating inter-patient variability in tumour size and morphology. Patient** a** presents a single nodular tumour, patient** b** presents a complex-shaped tumour, and patient** c** presents multifocal tumours of varying sizes. For each patient, a MRI with the liver and tumour VOIs, a CT image with the liver VOI on CT, and the associated SPECT image are shown. The liver VOIs are displayed in green and the tumour VOI in yellow

Of the 90 MRIs included in this dataset, 88 were already part of the ATLAS dataset [37]. Figure 1 illustrates the dataset.

The axial pixel spacing of the MR slices ranges from 0.68 mm $\times$ 0.68 mm to 1.41 mm $\times$ 1.41 mm, with a slice thickness between 2 mm and 4 mm. The axial pixel spacing of the CT images ranges from 0.94 mm $\times$ 0.94 mm to 0.98 mm $\times$ 0.98 mm, with a slice thickness between 1.25 mm and 2 mm. Finally, SPECT images were acquired using isotropic voxels of 4.4 mm.

SPECT images were resampled to a slice thickness of 2 mm using trilinear interpolation to match the slice thickness of the CT scanner that is associated with the SPECT/CT system.

### Manual and semi-automatic segmentation and registration

For SIRT treatment planning in our dataset, the entire liver and tumours are typically segmented manually on contrast-enhanced MRI, while the whole liver is delineated on the CT component of pre-treatment SPECT/CT using. Additional VOIs, such as the perfused liver or subregions defined by activity thresholds, can be generated to support planning decisions; however, these complementary segmentations are not systematic and were not considered in the present study. Although necrotic subregions are usually distinguished from viable tumour tissue and excluded from dosimetric evaluations, they were included here as part of the liver or tumour volumes, since they occurred in only a minority of cases. This choice was made to simplify implementation and ensure consistency across all pipelines.

In a second step, the liver contour delineated on MRI is registered onto the CT, allowing the transfer of tumour contours from MRI to CT space. This enables the estimation of the absorbed dose within the tumour using the intrinsic alignment between CT and SPECT images. Image registration was performed from the MRI scan, which was considered as the moving image, to the CT scan, which used as the fixed image. The process begins with a manual three-dimensional rigid alignment to establish an initial spatial correspondence. If greater accuracy is required, a non-rigid B-spline transformation is applied to capture local deformations. To further enhance the alignment, MIM’s Reg Refine tool can then be used, allowing manual placement of fixed points that are incorporated into the registration through a Gaussian mixture model, to improve local consistency. Once the deformable registration is complete, tumour contours delineated on the MRI can be transferred to the CT scan. These transferred contours can then be used for dosimetric evaluation within the tumour volume, taking advantage of the intrinsic alignment between the CT and SPECT images.

Liver and tumour volumes varied considerably across patients, reflecting inter-patient anatomical and pathological differences. On average, the liver volume was $1987 \pm 664$ cm^3^, while the tumour volume was $328 \pm 457$ cm^3^.

Inter-operator variability, estimated on a subset of 48 images as in [7], was assessed using independent segmentations performed by two experts and compared pairwise. The final segmentation used in this study on this subset was defined by expert consensus. Root mean square coefficients of variation were below $4.5\%$ for liver volumes and $6.2\%$ for tumour volumes.

### Automatic registration

Before performing automatic registration, MRI and CT images (with their associated VOIs) were resampled to an isotropic spacing of 1 mm. All images were normalised using intra-image min-max scaling. This normalisation was applied independently to each image to account for the strong inter-image intensity variability observed in MRI acquisitions. It also ensures comparable intensity ranges across modalities, which provides consistent inputs for the deep learning-based components of the pipeline. Liver CT volumes were automatically segmented using the 3D U-Net architecture SegmentationNet [38]. The MRI volumes were delineated either manually or automatically, depending on the configuration being tested.

Automatic registration was performed using the baseline proposed by Fragkiadakis et al. [10]. The process was carried out in two stages, global registration and local deformation, using the Elastix software [35]. Initially, the centre of gravity of the moving MRI liver mask was translated to match the centre of gravity of the fixed CT liver mask. Subsequently, the moving liver mask was aligned with the fixed liver mask through a 12-degree-of-freedom affine transformation. This alignment process was refined over four stages using a multiresolution registration approach based on a recursive image pyramid, which combines both down-sampling and Gaussian smoothing. The optimiser employed was the adaptive stochastic gradient descent, with the Dice coefficient serving as the similarity metric.

The affine transformation obtained at the end of the global registration process was used to initialise the local deformation step. Local deformation was performed using an intensity-based B-spline transformation, with binary masks employed to restrict the registration to specific regions of the image. The liver bounding boxes, derived from the automatic segmentations, served as the masks. Similar to the affine registration, a multiresolution strategy was applied, utilising a Gaussian smoothing image pyramid for down-sampling and refinement to ensure precise alignment. Adaptive stochastic gradient descent was employed as the optimiser with mutual information serving as the similarity metric.

After registration, the computed transformations were applied to the images at their native resolution. To evaluate the quality of the semi-automatic and automatic registration, the manual liver segmentation on the CT served as ground truth. Table 1 summarise the parameters used to perform automatic registration.

**Table 1:** Summary of Elastix parameters used for automatic MRI-to-CT registration

Reg stage	Role	Similarity metric	Multi-resolution	Control point
Affine	Global	Dice	Yes	Not applicable
Non-rigid B-spline	Local	Mattes Mutual Information	Yes	Coarse-to-fine grid

### Automatic segmentation

Automatic segmentation was conducted using two distinct approaches: monomodal segmentation (performed before registration) and multimodal segmentation (performed after registration). Both approaches followed the same segmentation process, differing only in the input data. In all automatic segmentation pipelines, the target output VOIs were the tumour and the whole liver. Monomodal segmentation used MRI data alone, whereas multimodal segmentation incorporated both MRI and SPECT data. The MRI was registered to the SPECT/CT acquisitions prior to segmentation. For monomodal pipelines, the ground truth corresponded to the original manual segmentations on the unregistered MRI, whereas for multimodal pipelines, it was derived from the registered MRI to ensure consistency with the input data.

The multiclass segmentation algorithm nnUNet [36] was selected to perform automatic segmentation due to its strong track record as a robust baseline model in the literature, particularly for the ATLAS dataset [39]. The models were trained for $2\,000$ epochs, each consisting of 250 mini-batches with a batch size of 2. The Adam optimiser [40] was employed, along with the Dice cross-entropy loss [41], which was calculated for both the non-tumoural liver and the tumour. For multimodal segmentation, two input channels were used, one for the MRI and one for the SPECT.

To prepare the training data, MRI and SPECT images, along with their associated VOIs, were resampled to the median MRI spacing of the dataset: 1.04mm $\times$ 1.04 mm $\times$ 3.0mm. The images were normalised using min-max scaling. During training, data augmentation techniques were applied, including random flips, rotations, intensity scaling, intensity shifting, contrast adjustments, and gamma correction. After each epoch, a validation metric was computed using an exponential moving average of the generalised Dice score for both the non-tumoural liver and the tumour.

A 5-fold nested cross-validation approach was employed. The dataset was randomly divided into five folds, each containing 18 images. Models were trained on three folds, validated on one fold, and tested on the remaining fold. This setup ensured that five models were trained, with each image appearing in the test set exactly once, allowing a comprehensive evaluation of the segmentation performance.

### Evaluation of different pipelines

To assess the impact of automatic registration and segmentation on dosimetry calculations, several processing pipelines were defined. The current clinical pretreatment workflow is referred to as the baseline pipeline. This pipeline uses the manual liver and tumour VOIs provided in the dataset, originally delineated on MRI by experienced radiologists. These MRI-derived VOIs are subsequently propagated to the CT images using semi-automatic MRI–CT registration. The baseline pipeline serves as a clinical reference for comparison with the other pipelines and is therefore treated as a ground truth, while acknowledging that it reflects current clinical practice rather than an absolute reference.

The pipeline 1 consists of the automatic registration of the liver from MRI to CT images helped by the manual segmentation of the liver on the MRI and the automatic liver segmentation on the CT. It allows us to evaluate the impact of the automatic registration alone on the absorbed dose calculation.

The pipeline 2 is similar to the pipeline 1. It consists of the automatic registration of the liver from the MRI to the CT but uses automatic liver segmentation on the MRI instead of manual segmentation. It allows us to evaluate the impact of using automatic liver segmentation on the automatic registration.

The pipeline 3 consists of performing automatic monomodal segmentation of the liver and tumour on the MRI followed by semi-automatic registration. This pipeline allows to measure the impact of automatic monomodal segmentation alone on absorbed dose calculation.

The pipeline 4 consists of automatic monomodal segmentation of the liver and tumour on the MRI but followed by an automatic registration. This pipeline allows to measure the combined impact of monomodal segmentation and automatic registration on absorbed dose calculation.

The pipeline 5 consists of semi-automatic registration of the MRI to the CT followed by automatic multimodal segmentation of the liver and tumour using the MRI and SPECT. This pipeline allows to quantify the impact of the SPECT on the automatic segmentation and the impact of multimodal segmentation on absorbed dose calculation.

Finally, the pipeline 6 is similar to pipeline 5 but with automatic registration. This registration is followed by an automatic multimodal segmentation of the liver and tumour using MRI and SPECT. This pipeline allows to measure the impact of automatic registration and automatic multimodal segmentation combined on absorbed dose calculation.

**Fig. 2:**
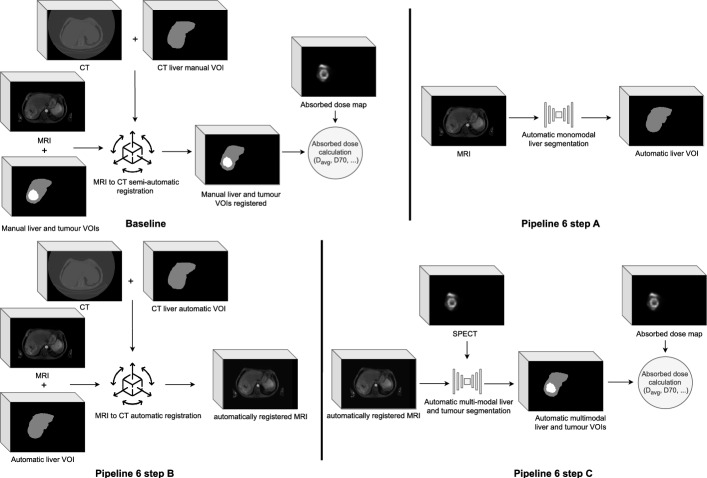
The steps involved in processing the data to obtain results for the baseline and pipeline number 6. The pipeline 6 is divided into three steps, first automatic liver VOIs are produced using a monomodal segmentation model (nnUNet) (step A). Then, using this VOI, MRI-to-CT automatic registration of the liver is performed (step B). Finally, a multimodal segmentation model is used to produce liver and tumour VOIs (nnUNet)(step C)

All pipelines are summarised in Table 2. The baseline pipeline and the pipeline number 6 are illustrated in Fig. 2.

**Table 2:** Synthesis of the segmentation and registration methods across the different pipelines, “seg” stands for segmentation and "reg" for registration

Pipeline	Segmentation method	Registration method
Baseline	Manual seg	Semi-automatic reg
Pipeline 1	Manual seg	Automatic reg using manual liver seg
Pipeline 2	Manual seg	Automatic reg using automatic liver seg
Pipeline 3	Automatic monomodal seg	Semi-automatic reg
Pipeline 4	Automatic monomodal seg	Automatic reg using automatic liver seg
Pipeline 5	Automatic multimodal seg	Semi-automatic reg
Pipeline 6	Automatic multimodal seg	Automatic reg using automatic liver seg

### Evaluation metrics

#### Registration and segmentation metrics

To evaluate the quality of liver and tumour registration and segmentation, several complementary metrics were computed: the Dice coefficient, Precision, Recall, the 95th percentile Hausdorff distance (HD95) [42] and the tumour involvement. These metrics provide a multifaceted assessment of both volumetric and surface agreement between the predicted and reference VOIs.

1. **Dice coefficient:** This metric evaluates the volumetric overlap between two binary masks. In this study, the Dice coefficient provides a first-order indicator of anatomical agreement and is used to relate geometric accuracy to dosimetric accuracy. For a given class and voxel-wise comparison, it is defined as:
  1 $$\begin{aligned} \text {Dice} = \frac{2 \times \text {TP}}{\text {TP} + \text {FP} + \text {FN}}, \end{aligned}$$
  where TP, FP, and FN are the numbers of true positive, false positive, and false negative voxels, respectively.
2. **Precision:** This metric assesses how many of the predicted positive voxels are truly relevant. In the context of dosimetry, high precision is particularly important to avoid the inclusion of non-tumoural tissue in tumour VOIs, which could lead to dose underestimation.
  2 $$\begin{aligned} \text {Precision} = \frac{\text {TP}}{\text {TP} + \text {FP}}. \end{aligned}$$
3. **Recall:** Also referred to as sensitivity, this metric evaluates how many true positive voxels are correctly detected. This metric is critical in our study to ensure that tumour regions are not missed, as under-segmentation may result in substantial underestimation of absorbed dose.
  3 $$\begin{aligned} \text {Recall} = \frac{\text {TP}}{\text {TP} + \text {FN}}. \end{aligned}$$
4. **HD95:** HD95 captures the 95th percentile of the maximum distances between surfaces. HD95 provides a robust measure of boundary alignment while limiting the influence of extreme outliers that are less relevant for dosimetric impact.
  4 $$\begin{aligned} \text {HD95}(A, B) = \max \left( \text {P}_{95} \left( \min _{b \in B} d(a, b) \right) _{a \in A}, \text {P}_{95} \left( \min _{a \in A} d(b, a) \right) _{b \in B} \right) , \end{aligned}$$
  where *d*(*a*, *b*) is the Euclidean distance between points *a* and *b* from point sets *A* and *B*. and $\text {P}_{95}$ represents the 95^th^ percentile of the minimum distances.
5. **Tumour involvement:** this metric quantifies the proportion of tumour tissue relative to the total segmented liver volume. In this work, tumour involvement is used to assess systematic volumetric biases introduced by automatic methods and to interpret their impact on absorbed dose estimates.
  5 $$\begin{aligned} \text {Tumour involvement} = \frac{\text {Number of tumour voxels}}{\text {Number of liver voxels}}, \end{aligned}$$

For registration (baseline, pipeline 1 and pipeline 2), these metrics were first calculated between the registered MRI liver and the liver delineated on the CT. Secondly, they were computed between the liver and tumours obtained through semi-automatic registration and automatic registration together.

For segmentation (pipeline 3 to 6), the metrics were computed by comparing automatic segmentations with manually delineated liver and tumour VOIs on MRI (before or after registration, depending on the pipeline).

#### Dosimetry comparison metrics

To perform the dosimetry calculations, all VOIs (semi-automatically or automatically registered, and manually or automatically segmented) were resampled to match the original spacing of the SPECT images. Assuming that the $^{99\text {m}}$Tc activity distribution accurately represents that of ^90^Y, SPECT images were converted from counts to absorbed dose maps using the local deposition method (LDM) [43]. In clinical practice, a fixed activity of 150 MBq of $^{99\text {m}}$Tc-MAA is administered to all patients during the pre-treatment phase. Then, all counts in the $^{99\text {m}}$Tc-MAA SPECT image are normalised to an intended ^90^Y prescribed activity of 1 GBq using the patient-relative conversion method described in [3]. This process was implemented in Python.

For all volumes of interest, the average absorbed dose ($D_{avg}$) and maximum absorbed dose were calculated ($D_{max}$). Additionally, for the tumour, the normalised cumulative dose-volume histogram (cDVH) was computed. Both dose and volume were normalised between 0 and 1. From the cDVH, the minimum dose to 70% received by of the volume (D70) was extracted. Furthermore, the inverse of the cDVH, denoted $\text {cDVH}^{-1}$, was also calculated. It can be expressed as:

6 $$\begin{aligned} \text {cDVH}^{-1}(\nu ) = \sup \{ D \in \mathbb {R}, \text {cDVH}(D) \ge \nu \}, \end{aligned}$$

where $\nu$ is the cumulative volume fraction, representing the volume that has received at least the given dose *D*. $\text {cDVH}^{-1}$ comparison were performed by computing the L1 distance between the baseline ($\text {cDVH}^{-1}_{\text {bs}}$) and the proposed pipelines ($\text {cDVH}^{-1}_{\text {pr}}$). This metric allows to express the differences between two $\text {cDVHs}^{-1}$ relatively to the maximum dose measured in a voxel.

7 $$\begin{aligned} L^1(\text {cDVH}^{-1}_{\text {bs}}, \text {cDVH}^{-1}_{\text {pr}}) = \int _{0}^{1} \left| \text {cDVH}^{-1}_{\text {bs}}(\nu ) - \text {cDVH}^{-1}_{\text {pr}}(\nu ) \right| \, d\nu \end{aligned}$$

Four patients with multifocal tumours were excluded from the dosimetry calculation as only one tumour was treated, and partial $^{99\text {m}}$Tc-MAA injections could skew the results. This exclusion does not impact segmentation, which detects all tumours, but ensures dosimetry focuses on treated tumours for accurate absorbed dose calculation.

All dosimetric calculations,including absorbed dose maps, D70 values, and dose-volume histograms (DVHs) were performed using Python.

#### Statistical analysis

Finally, to assess the statistical significance of the results between pipelines, a Shapiro–Wilk test was initially performed to determine whether the data followed a normal distribution. Based on the outcome, either a paired t-test (for normally distributed data) or a paired Wilcoxon test (for non-normally distributed data) was applied. Statistical significance was interpreted using a conventional threshold: a p-value less than 0.05 was considered statistically significant.

## Results

### Registration and segmentation performance metrics

#### Impact of the registration

The MRI-to-CT registration results for the baseline, pipeline 1, and pipeline 2 are presented in Table 3. The baseline relies on the semi-automatic clinical registration, whereas pipeline 1 and pipeline 2 use fully automatic registration based on manual and automatic liver segmentation, respectively. Regardless of the registration method, the results were highly similar, with differences in the Dice coefficient of less than $1\%$ and standard deviations below $3\%$. The baseline pipeline showed slightly higher Dice values ($91.9\%$) compared to pipeline 1 ($91.7\%$) and pipeline 2 ($91.3\%$), while underperforming in terms of Precision and HD95. Additionally, the baseline exhibited a higher standard deviation compared to pipelines 1 and 2 for all metrics.

Despite these very small absolute differences, paired statistical analysis revealed that the Dice score was significantly lower for both pipeline 1 ($p = 0.002$) and pipeline 2 ($p < 0.00003$) when compared with the baseline, indicating that these differences, although limited in magnitude, were consistently observed across patients.

When comparing pipeline 1 to pipeline 2, no statistically significant differences were observed, indicating that replacing manual liver segmentation with automatic liver segmentation had no measurable impact on registration performance.

**Table 3:** MRI-to-CT liver registration performances across the entire dataset

Pipeline	Dice (%) $\uparrow$	Precision (%) $\uparrow$	Recall (%) $\uparrow$	HD95 (mm) $\downarrow$
Baseline	91.9 ± 2.5	90.0 ± 3.6	*94.0* ± 2.9	8.2 ± 3.4
Pipeline 1	91.7 ± 1.9	91.1 ± 2.8	92.3 ± 2.5	7.6 ± 2.2
Pipeline 2	91.3 ± 1.9	90.7 ± 2.9	92.1 ± 2.6	8.0 ± 2.6

We can also measure the alignment between livers and tumours semi-automatically (baseline) and automatically registered (pipeline 1 and 2). Tables 4 and 5 report the results for the liver and the tumour, respectively.

When comparing the manual segmentations after semi-automatic (baseline) and automatic registration (pipelines 1 and 2) on the liver, Dice scores, Precision, and Recall range between 90.5 and $94.2\%$.

However, when focussing on tumour performance, a significant discrepancy is observed. The Dice score is approximately $79\%$, and the other geometric metrics range between 78.5 and $79.9\%$.

**Table 4:** Average liver registration performance between the manually segmented MRIs automatically registered by pipelines 1 and 2, and the manually segmented MRIs semi-automatically registered, across the full dataset

Pipeline	Dice $\uparrow$	Precision $\uparrow$	Recall $\uparrow$	HD95 (mm) $\downarrow$
pipeline 1	92.4 ± 2.6	93.9 ± 3.3	91.2 ± 3.4	6.5 ± 3.2
pipeline 2	92.6 ± 2.6	94.2 ± 3.1	91.2 ± 3.3	6.4 ± 3.0

**Table 5:** Average tumour registration performance between the manually segmented MRIs automatically registered by pipelines 1 and 2, and the manually segmented MRIs semi-automatically registered, across the full dataset

Pipeline	Dice $\uparrow$	Precision $\uparrow$	Recall $\uparrow$	HD95 (mm) $\downarrow$
pipeline 1	79.0 ± 15.3	78.5 ± 16.0	79.9 ± 15.4	6.7 ± 3.2
pipeline 2	78.9 ± 15.5	78.5 ± 16.3	79.7 ± 15.7	6.6 ± 3.1

**Fig. 3:**
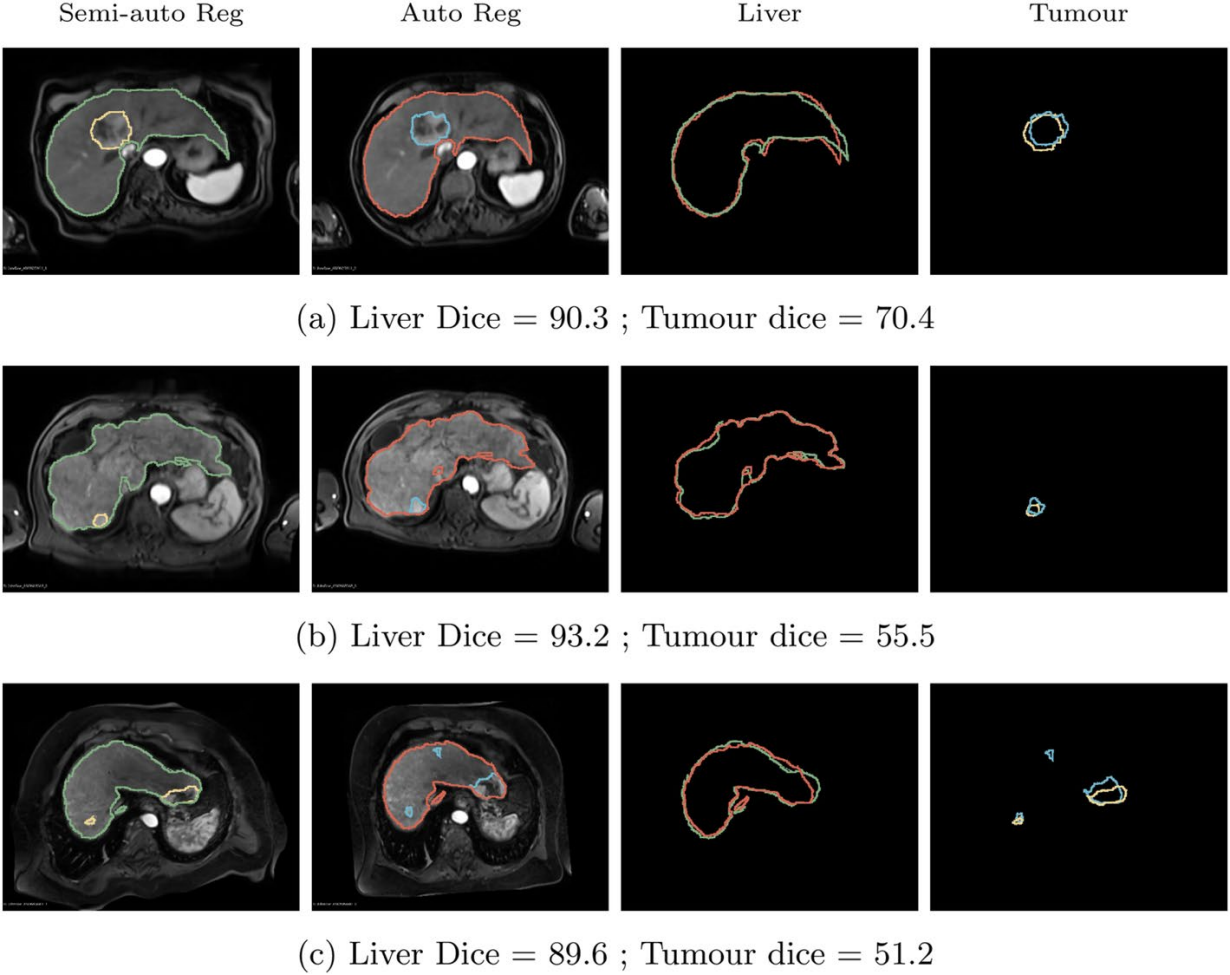
Axial slices from three representative patients illustrating differences between semi-automatic (baseline) and automatic (pipeline 1) registration. For each patient, the liver and tumour contours obtained using the baseline semi-automatic registration are shown, along with the corresponding contours obtained using automatic registration (pipeline 1). In the semi-automatic registration, the liver is displayed in green and the tumour in yellow, whereas in the automatic registration the liver is displayed in red and the tumour in blue

Figure 3 provides a qualitative illustration of the limitation of registration to accurately capture local tumour alignment when global organ alignment is satisfactory. Three representative patient cases are shown, in which liver Dice between the baseline and pipeline 1 achieves Dice scores close to $90\%$, while tumour Dice remains substantially lower, with Dice values ranging from 51.2 to $70.4\%$. These examples illustrate that, due to the large difference in structure size, an accurate global liver alignment does not necessarily translate into an accurate local alignment of small tumour regions. In such cases, local deformations affecting the tumour are insufficiently modelled, leading to local misalignments despite good overall liver registration.

Finally, a comparison of tumour involvement across the baseline and the two registration-only pipelines (1 and 2) is presented in Table 6. Overall, only slight differences are observed between the measured values. These discrepancies may result from the baseline registration applying broader or less constrained deformations, while pipelines 1 and 2 use more refined or regularised transformations.

**Table 6:** Average tumour involvement (%) measured for the baseline and registration-only pipelines

Pipeline	Tumour involvement (%)
Baseline	12.9 ± 14.2
pipeline 1	14.1 ± 14.4
pipeline 2	14.2 ± 14.5

#### Impact of the segmentation

Tables 7 and 8 report the segmentation results for pipelines involving an automatic segmentation step (pipelines 3 to 6) with equivalent registration, using the manual segmentation on the MRI images as ground truth.

All approaches deliver a comparable level of performance for liver segmentation, with Dice scores exceeding $94.8\%$. In contrast, tumour segmentation shows substantially lower performance across all metrics, with Dice scores barely exceeding $60\%$. All models exhibit high inter-patient variability, as evidenced by the large standard deviations. However, a performance difference is observed when comparing pipelines 3 and 4 to pipeline 6, which achieves better results across all computed metrics, including a 4.1 points of percentage improvement in the Dice coefficient, increasing from 62.5 to $66.6\%$.

**Table 7:** Average liver segmentation performance for pipelines 3 to 6 involving automatic segmentation

Pipeline	Dice $\uparrow$	Precision $\uparrow$	Recall $\uparrow$	HD95 (mm) $\downarrow$
pipeline 3 & 4	94.8 ± 2.5	95.5 ± 2.8	94.2 ± 3.9	6.1 ± 5.5
pipeline 5	94.9 ± 2.2	95.1 ± 2.7	94.8 ± 3.0	5.8 ± 4.0
pipeline 6	95.1 ± 1.7	95.9 ± 2.6	94.4 ± 2.8	5.6 ± 3.9

These metrics were computed over the entire dataset, using manual segmentations on MRI as ground truth

**Table 8:** Average tumour segmentation performance for pipelines 3 to 6 involving automatic segmentation

Pipeline	Dice $\uparrow$	Precision $\uparrow$	Recall $\uparrow$	HD95 (mm) $\downarrow$
pipeline 3 & 4	62.5 ± 28.4	74.7 ± 28.3	60.2 ± 30.2	40.6 ± 61.3
pipeline 5	64.5 ± 27.2	78.8 ± 22.8	62.2 ± 29.4	24.9 ± 23.3
pipeline 6	66.6 ± 24.7	74.8 ± 24.3	66.9 ± 25.3	24.5 ± 21.5

These metrics were computed over the entire dataset, using manual segmentations on MRI as ground truth

**Fig. 4:**
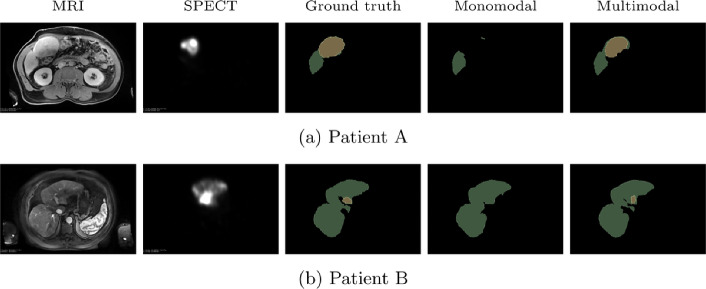
Comparison of automatic tumour segmentations obtained with monomodal (pipeline 3) and multimodal (pipeline 5) approaches. Two representative patients are shown, with the MRI, SPECT, ground truth, monomodal and multimodal segmentations

Figure 4 illustrates the improvement achieved by incorporating multimodal data compared to monomodal data for automatic segmentation with same semi-automatic registration, by comparing the results obtained with pipelines 3 and 5. In both presented cases, the monomodal model (pipeline 3) fails to correctly classify any voxels as tumour, whereas the multimodal model (pipeline 5) successfully identifies a substantial portion of the tumour. This segmentation is notably closer to the ground truth provided by the baseline, demonstrating the added value of SPECT information for segmentation.

Finally, Table 9 provides a comparison of tumour involvement across the baseline and pipelines 3 to 6. Overall, all automatic segmentation pipelines tend to underestimate the tumour involvement compared to the baseline. This suggests that automatic models generally predict smaller tumour volumes than those actually present. Among these, pipeline 6, which combines multimodal inputs with full automation, shows the smallest discrepancy, yielding a tumour involvement estimate closer to the baseline reference.

**Table 9:** Average tumour involvement (%) measured for the baseline and the automatic segmentation pipelines (3 to 6)

Pipeline	Tumour involvement (%)
Baseline	12.9 ± 14.2
Pipeline 3	10.5 ± 12.3
Pipeline 4	10.8 ± 12.5
Pipeline 5	10.4 ± 11.1
Pipeline 6	12.0 ± 11.8

### Dosimetry performance metrics

The dosimetry results calculated for each pipeline are presented in Table 10. The baseline showed a $D_{avg}$ of 15.9 Gy for the non-tumoural liver, with a substantial standard deviation of 49.6 Gy. For the tested pipelines, the differences to the baseline pipeline in $D_{avg}$ for the non-tumoural liver were minimal, with average variations below 1 Gy.

For the tumour, the baseline exhibited significantly higher $D_{avg}$ values, with an average of 115.6 Gy and a standard deviation of 106.7 Gy. As expected, the different pipelines showed more pronounced differences in tumour dosimetry than in non-tumoural liver, with $D_{avg}$ variations from the baseline ranging from $-17.1$ Gy to 14.9 Gy.

Regarding the L1 norm between the $\text {cDVH}^{-1}$ of the baseline and the pipelines, pipelines 1 and 2, which involved only registration, showed a L1 distance of approximately $350\%$ of the $D_{max}$, with standard deviations around $450\%$. Pipelines involving automatic segmentation (3 to 6) exhibited larger L1 norms, ranging from $566.2\%$ in pipeline 6 to $703.6\%$ in pipeline 4. Monomodal segmentation pipelines (3 and 4) demonstrated greater discrepancies than multimodal segmentation pipelines (5 and 6), especially in terms of standard deviation, which reached $1001\%$ in pipeline 4 compared to $566.2\%$ in pipeline 6.

Additionally, monomodal pipelines 3 and 4 tended to underestimate the average absorbed dose and D70, whereas multimodal pipelines 5 and 6 showed a tendency to overestimate these values. This highlights the influence of segmentation strategy on the dosimetry outcomes.

**Table 10:** Average dosimetry calculation across the entire dataset in Gy

Average values (Gy)					
Pipeline	$D_{avg}$ Whole Liver	$D_{avg}$ Non-tumoural Liver	$D_{avg}$ Tumour	D70 Tumour	_
baseline	22.2 ± 49.6	15.9 ± 35.3	115.6 ± 106.7	70.6 ± 66.0	_

Differences with Baseline (Gy)					
Pipeline	$D_{avg}$ Whole Liver	$D_{avg}$ Non-tumoural Liver	$D_{avg}$ Tumour	D70 Tumour	Tumour L1 (%)
pipeline 1	− 0.7 ± 1.7	− 1.1 ± 1.6	− 8.8 ± 57.7	− 9.8 ± 54.8	349.6 ± 442.7
pipeline 2	− 0.8 ± 1.7	− 1.1 ± 1.7	− 9.2 ± 56.9	− 10.2 ± 54.2	359.2 ± 454.2
pipeline 3	− 0.1 ± 1.8	1.8 ± 3.4	− 13.2 ± 64.3	− 9.4 ± 61.5	637.6 ± 969.5
pipeline 4	0.2 ± 1.9	2.1 ± 3.4	− 17.1 ± 71.2	− 13.8 ± 58.9	703.6 ± 1001.0
pipeline 5	0.1 ± 0.9	0.4 ± 3.5	14.9 ± 52.6	14.3 ± 45.2	607.1 ± 737.0
pipeline 6	0.6 ± 1.5	0.3 ± 3.8	6.6 ± 56.8	3.5 ± 53.2	566.2 ± 649.4

The first row contains the baseline average values, while the rows below correspond to the mean of intra-patient differences with respect to the baseline. The last column is the L1 norm between the $\text {cDVH}^{-1}$ of pipelines and the baseline over the tumour expressed in percentage of the $D_{max}$

**Fig. 5:**
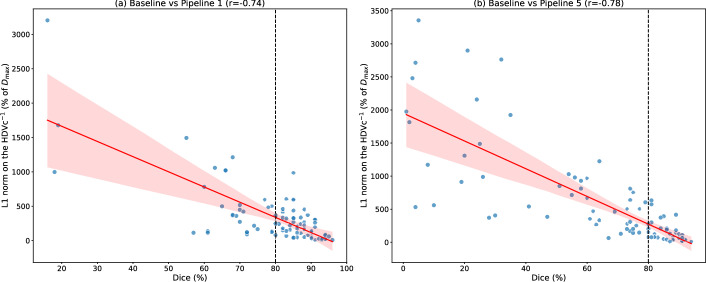
Relationship between the Dice coefficient and the L1 norm on $\text {cDVH}^{-1}$ for the tumour, comparing the baseline with pipeline 1 and pipeline 5. Each point corresponds to a patient, and the L1 norm quantifies the dosimetric difference between the $\text {cDVH}^{-1}$ curves. The red line represents the fitted linear regression, and *r* denotes the Pearson correlation coefficient. The black dashed vertical line indicates the Dice score cutoff at $80\%$.

Figure 5 presents the relative differences per patient for the L1 norm computed between the $\text {cDVH}^{-1}$ of the baseline and pipeline 1 and 5. This comparison allows the dosimetric impact to be assessed either at same segmentation when only the registration differs (pipeline 1 vs baseline), or at same registration when using multimodal tumour segmentation (pipeline 5 vs baseline). A clear negative correlation is observed in both cases between the Dice coefficient and the L1 norm, with Pearson correlation coefficients of $r = -0.74$ and $r = -0.78$, respectively. This indicates that when either only the registration or only the segmentation is automated, patients with higher Dice scores tend to exhibit smaller dosimetric differences on the $\text {cDVH}^{-1}$ curves.

Registration-only differences between the baseline pipeline and pipeline 1 had a significant impact on the predicted doses. On average, a $21\%$ difference in tumour Dice resulted in statistically significant variations in dose estimates, with p-values close to zero ($10^{-8}$). Although the average dosimetric difference was small, the dose calculated with the baseline was almost systematically higher than that obtained with pipeline 1.

A similar analysis was performed to measure the segmentation only differences between the baseline and pipeline 5 on the per-patient paired differences in $D_{\text {avg}}$ and $D_{70}$. While significant differences were found when considering all cases, these were no longer statistically significant when restricting the analysis to the 36 cases with a Dice score of at least $80\%$ between segmentations (p-values of 0.084 and 0.055 for $D_{\text {avg}}$ and $D_{70}$, respectively).

## Discussion

### Automatic registration impact

Evaluating automatic registration poses significant challenges. According to the values in Table 3, no substantial differences were observed when comparing semi-automatic (baseline) and automatic registrations (pipelines 1 and 2) of MRI-to-CT. However, notable differences became apparent when our metrics were computed directly between the segmentation of the MRI with semi-automatic and automatic registrations (Tables 4 and 5). Differences in Dice values were substantial for liver segmentation ($7.5\%$ on average); however, the greatest impact was observed in tumour segmentation ($21\%$) leading to significant variations in the predicted doses, as confirmed by a Wilcoxon signed-rank test.

From a dosimetric perspective, as illustrated by Table 10 and Fig. 5, the comparison between the baseline pipeline and the pipelines 1 and 2 shows that most cases exhibit high Dice coefficients, which correspond to small dosimetric discrepancies. This suggests that, in general, minor differences in alignment between semi-automatic and automatic registration do not substantially impact the estimated dose. However, in certain cases, small misalignments at the liver level can spread to the tumour region, particularly when the tumour is small. This may result in a substantial drop in the Dice score and lead to large differences in the absorbed dose.

Nevertheless, since the tumour ground truth on the CT is unavailable and the liver performances are similar, it remains inconclusive which registration method performs better on the tumour. Drawing such a conclusion would require a substantial number of CT scans in which the tumour is clearly visible. Two possible solutions can be considered to overcome this limitation: the first is to assemble a dataset with a sufficiently large number of CT scans where the tumours are clearly visible, in order to properly evaluate tumour registration. Alternatively, one could favour a pre-treatment acquisition protocol using SPECT / contrast-enhanced CT imaging, which would allow for direct tumour segmentation on the CT and thus eliminate the need for registration.

This impact of small misalignments on dosimetry has also been reported in the literature. Tang et al. [15] investigated the role of registration strategies in SIRT dosimetry by introducing segmentation-guided and landmark-guided multimodal registration methods to align MRI and CT images with the non-contrast-enhanced CT used for dose calculation. In a study involving 20 patients, they demonstrated that incorporating CNN-based liver segmentations and anatomical landmarks significantly improved alignment quality. Most notably, even when global registration metrics appeared similar, small misalignments in tumour localisation resulted in dose differences exceeding $10\%$ in some cases aligning with our findings.

### Automatic segmentation pipelines comparison

As observed in previous studies [39], our results show a significant inter-patient variability in tumour segmentation performance. While models achieved Dice scores above $80\%$ for $40\%$ of patients and between $60\%$ and $80\%$ in the $30\%$ of the cases, the remaining $30\%$ remains challenging to segment. In some cases, the Dice scores were as low as $0\%$, indicating that no pixels were classified as tumour.

A qualitative review of the most challenging cases, corresponding to approximately $30\%$ of patients with Dice scores below $60\%$, highlights several recurring tumour characteristics. First, very small tumours are particularly difficult to segment accurately, as the misclassification of only a few voxels can lead to a large relative error. Second, large tumours with complex or irregular shapes are often only partially segmented: in these cases, the model typically identifies the most prominent tumoural region but fails to accurately delineate the tumour boundaries, leading to systematic under-segmentation at the edges. Third, in patients with multifocal tumours, the model generally succeeds in detecting the main lesion but tends to miss smaller satellite tumours. Finally, tumours exhibiting atypical texture or contrast patterns can also challenge the model.

For both pipelines 5 and 6, incorporating SPECT images does not improve liver segmentation, as expected, given that the injection targets the tumoural area. However, it does enhance tumour segmentation performance compared to pipelines 3 and 4, which were performed without SPECT data (Table 8). This improvement is even more pronounced in HD95, with a decrease of 16.1 mm, when comparing pipelines 3 and 4 with pipeline 6. As illustrated in Fig. 4, multimodal models demonstrate a clear advantage in cases where monomodal approaches fail to detect the tumour. While pipelines 3 and 4 performing segmentation without SPECT often misses the tumour entirely, the inclusion of SPECT systematically enables the model to identify tumoural regions. These improvements are also visible in the L1 norm on $\text {cDVH}^{-1}$ (Table  10), which not only shows smaller values but also reduced standard deviations, indicating greater stability in absorbed dose predictions with multimodal segmentation. These gains are particularly noticeable in challenging cases with low monomodal performance. pipeline 6 systematically addresses situations where other automatic pipelines fail to classify any pixels as tumour, thereby enhancing the performance in complex cases. Although the average difference in tumour absorbed dose observed for pipeline 6, which relies on multimodal automatic processing, remains limited (6.6 Gy) according to the Table 10, the associated standard deviation is large (56.8 Gy), indicating substantial variability across patients.

The benefit of multimodal segmentation has also been reported in the literature. Jafargholi Rangraz et al. [16] proposed a multimodal pipeline combining pre-therapeutic MAA SPECT/CT, contrast-enhanced CBCT, and ^18^F-FDG PET/CT for liver and tumour segmentation in voxel-based dosimetry. After rigid and deformable registration into a common space, semi-automatic liver and perfusion territory segmentations showed high agreement with expert contours (Dice coefficient of $92\%$). These results support the use of multimodal segmentation for accurate pre-treatment dosimetry when single-modality delineation is limited.

Based on these findings, the automation of liver segmentation can reasonably be considered in the short term, as its impact on dosimetry remains limited. In contrast, tumour segmentation remains more challenging. While automatic methods perform well for tumours with simple shapes and homogeneous appearance, their robustness decreases in the presence of complex morphologies, heterogeneous uptake, or multifocal lesions. In such cases, automatic segmentation should be regarded as an assistive tool rather than a definitive solution, and expert validation remains essential. The integration of uncertainty maps alongside segmentation outputs could further facilitate the clinical adoption of these models. By highlighting areas where the confidence of the model is low, such maps would provide valuable guidance to clinicians, enabling them to better assess the reliability of automated predictions and focus their attention on regions requiring expert validation.

In a pre-treatment setting involving SPECT imaging, the ideal scenario would include contrast-enhanced CT, as it provide complementary anatomical and functional information for accurate tumour delineation. In practice, tumour segmentation might be performed directly on the contrast-enhanced CT. Still, segmentation performance and the underlying technologies used for automation on MRI and CT are relatively similar, meaning that the challenges previously discussed for MRI-based segmentation would also directly apply to CT-based segmentation [11].

## Conclusion

The study evaluated the impact of automatic registration and segmentation on absorbed dose calculations in SIRT treatment planning. While automatic liver registration yields liver Dice scores comparable to semi-automatic methods, significant differences are observed in the predicted absorbed tumour doses between the two approaches. However, as tumours were not visible on CT scans, it remains inconclusive which method is superior.

For segmentation, a correlation between Dice scores and measured absorbed tumour doses underscores the importance of improving accuracy. Multimodal data integration enhances performance, with potential gains of up to 4.1 points of percentage. For tumour segmentation, despite advancements, current accuracy remains insufficient for full automation. Achieving a consistent Dice score above $80\%$ appears to be a necessary criterion for automation. Currently, this benchmark is met in $40\%$ of cases, with an additional $30\%$ showing promising progress. However, the remaining $30\%$ represents the most significant challenge to address.

In addition, the clinical utility of automated versus manual dosimetric evaluation is inherently influenced by tumour-specific characteristics and heterogeneous uptake patterns, as well as by inter-patient differences in arterial perfusion and systemic behaviour. These factors suggest that the choice between manual and automated registration / segmentation strategies may need to be adapted on a case-by-case basis. In practice, this decision is likely to rely on an initial assessment of the available imaging modalities, including CT, MRI, and SPECT/CT, to determine whether automated methods can be applied reliably or whether expert-driven approaches remain necessary.

## Data Availability

The dataset used in this study is partially available as part of the ATLAS dataset [37]. It has been fully anonymised.

## References

[1] Bray F, Laversanne M, Sung H, Ferlay J, Siegel RL, Soerjomataram I, et al. Global cancer statistics 2022: GLOBOCAN estimates of incidence and mortality worldwide for 36 cancers in 185 countries. CA Cancer J Clin. 2024;74(3):229–63. 10.3322/caac.21834. 38572751 10.3322/caac.21834

[2] Galle PR, Forner A, Llovet JM, Mazzaferro V, Piscaglia F, Raoul JL, et al. EASL clinical practice guidelines: management of hepatocellular carcinoma. J Hepatol. 2018;69(1):182–236. 10.1016/J.JHEP.2018.03.019. 29628281 10.1016/j.jhep.2018.03.019

[3] Chiesa C, Sjogreen-Gleisner K, Walrand S, Strigari L, Flux G, Gear J, et al. EANM dosimetry committee series on standard operational procedures: a unified methodology for 99mTc-MAA pre- and 90Y peri-therapy dosimetry in liver radioembolization with 90Y microspheres. Eur J Nucl Med Mol Imaging. 2021;8(77):1–44. 10.1186/s40658-021-00394-3. 10.1186/s40658-021-00394-3 PMC8589932 34767102

[4] Gnesin S, Canetti L, Adib S, Cherbuin N, Monteiro MS, Bize P, et al. Partition model-based 99mTc-MAA SPECT/CT predictive dosimetry compared with 90Y TOF PET/CT posttreatment dosimetry in radioembolization of hepatocellular carcinoma: a quantitative agreement comparison. J Nucl Med. 2016;57(11):1672–8. 27307346 10.2967/jnumed.116.173104

[5] Jadoul A, Bernard C, Lovinfosse P, Gérard L, Lilet H, Cornet O, et al. Comparative dosimetry between 99m Tc-MAA SPECT/CT and 90 Y PET/CT in primary and metastatic liver tumors. Eur J Nucl Med Mol Imaging. 2020;47:828–37. 31388721 10.1007/s00259-019-04465-7

[6] Weber M, Lam M, Chiesa C, Konijnenberg M, Cremonesi M, Flamen P, et al. EANM procedure guideline for the treatment of liver cancer and liver metastases with intra-arterial radioactive compounds. Eur J Nucl Med Mol Imaging. 2022;49(5):1682–99. 10.1007/s00259-021-05600-z. 35146577 10.1007/s00259-021-05600-z PMC8940802

[7] Nodari G, Popoff R, Riedinger J, Lopez O, Pellegrinelli J, Dygai-Cochet I, et al. Impact of contouring methods on pre-treatment and post-treatment dosimetry for the prediction of tumor control and survival in HCC patients treated with selective internal radiation therapy. Eur J Nucl Med Mol Imaging Res. 2021. 10.1007/s00259-020-04988-4. 10.1186/s13550-021-00766-x PMC7943673 33687596

[8] Lange T, Wenckebach TH, Lamecker H, Seebass M, Hünerbein M, Eulenstein S, et al. Registration of different phases of contrast-enhanced CT/MRI data for computer-assisted liver surgery planning: evaluation of state-of-the-art methods. Int J Med Robotics + Comp Assist Surg MRCAS. 2005;1(3):6–20. 10.1002/RCS.23. 10.1002/rcs.23 17518386

[9] Zhou B, Augenfeld Z, Chapiro J, Zhou SK, Liu C, Duncan JS. Anatomy-guided multimodal registration by learning segmentation without ground truth: Application to intraprocedural CBCT/MR liver segmentation and registration. Med Image Anal. 2021;71:102041. 33823397 10.1016/j.media.2021.102041 PMC8184611

[10] Fragkiadakis A, Quinton F, Popoff R, Nodari G, Lopez O, Pellegrinelli J, et al. CT-MRI liver registration for selective internal radiation therapy. In: SPIE Medical Imaging 2024: Image Processing. vol. 12926. SPIE; 2024. p. 438–447.

[11] Bilic P, Christ P, Li HB, Vorontsov E, Ben-Cohen A, Kaissis G, et al. The liver tumor segmentation benchmark (lits). Med Image Anal. 2023;84:102680. 36481607 10.1016/j.media.2022.102680 PMC10631490

[12] Survarachakan S, Prasad PJR, Naseem R, de Frutos JP, Kumar RP, Langø T, et al. Deep learning for image-based liver analysis-A comprehensive review focusing on malignant lesions. Artif Intell Med. 2022;130:102331. 35809970 10.1016/j.artmed.2022.102331

[13] Li J, Anne R. Evaluation of Atlas-based auto-segmentation of liver in MR images for Yttrium-90 selective internal radiation therapy. J Appl Clin Med Phys. 2023;24(5):e13979. 37070130 10.1002/acm2.13979 PMC10161143

[14] Luu MH, Mai HS, Pham XL, Le QA, Le QK, Van Walsum T, et al. Quantification of liver-Lung shunt fraction on 3D SPECT/CT images for selective internal radiation therapy of liver cancer using CNN-based segmentations and non-rigid registration. Comput Methods Programs Biomed. 2023;233:107453. 36921463 10.1016/j.cmpb.2023.107453

[15] Tang X, Jafargholi Rangraz E, Heeren R, Coudyzer W, Maleux G, Baete K, et al. Segmentation-guided multi-modal registration of liver images for dose estimation in SIRT. Eur J Nucl Med Mol Imaging Phys. 2022. 10.1186/s40658-022-00432-8. 10.1186/s40658-022-00432-8 PMC8790002 35076801

[16] Jafargholi Rangraz E, Coudyzer W, Maleux G, Baete K, Deroose CM, Nuyts J. Multi-modal image analysis for semi-automatic segmentation of the total liver and liver arterial perfusion territories for radioembolization. Eur J Nucl Med Mol Imaging Res. 2019. 10.1186/S13550-019-0485-X. 10.1186/s13550-019-0485-x PMC6382918 30788640

[17] Stella M, van Rooij R, Lam MGEH, de Jong HWAM, Braat AJAT. Automatic healthy liver segmentation for holmium-166 radioembolization dosimetry. EJNMMI Res. 2023;13(1):68. 37453996 10.1186/s13550-023-00996-1 PMC10349793

[18] Rahimi A, Khalil A, Faisal A, Lai KW. CT-MRI Dual information registration for the diagnosis of liver cancer: a pilot study using point-based registration. Curr Med Imaging. 2022;18(1):61–6. 10.2174/1573405617666210825155659. 34433403 10.2174/1573405617666210825155659

[19] Foruzan AH, Motlagh HR. Multimodality liver registration of Open-MR and CT scans. Int J Comput Assist Radiol Surg. 2015;10(8):1253–67. 10.1007/S11548-014-1139-0. 25556525 10.1007/s11548-014-1139-0

[20] Viola P, Wells WM. Alignment by Maximization of Mutual Information. Int J Comput Vision. 1997;24(2):137–54. 10.1023/A:1007958904918/METRICS.

[21] Rueckert D, Sonoda LI, Hayes C, Hill DLG, Leach MO, Hawkes DJ. Nonrigid registration using free-form deformations: application to breast MR images. IEEE Trans Med Imaging. 1999;18(8):712–21. 10534053 10.1109/42.796284

[22] Cahill ND, Noble JA, Hawkes DJ, Demons algorithms for fluid and curvature registration. In: Proceedings - 2009 IEEE International Symposium on Biomedical Imaging: From Nano to Macro. ISBI. 2009;730–3. 10.1109/ISBI.2009.5193151.

[23] Thevenaz P, Ruttimann UE, Unser M. A pyramid approach to subpixel registration based on intensity. IEEE Trans Image Process. 1998;7(1):27–41. 18267377 10.1109/83.650848

[24] Liao R, Miao S, De Tournemire P, Grbic S, Kamen A, Mansi T, et al. An Artificial Agent for Robust Image Registration;. Available from: www.aaai.org.

[25] Hu Y, Modat M, Gibson E, Li W, Ghavami N, Bonmati E, et al. Weakly-supervised convolutional neural networks for multimodal image registration. Med Image Anal. 2018;10(49):1–13. 10.1016/J.MEDIA.2018.07.002. 10.1016/j.media.2018.07.002 PMC6742510 30007253

[26] Cao X, Yang J, Wang L, Xue Z, Wang Q, Shen D. Deep Learning based Inter-Modality Image Registration Supervised by Intra-Modality Similarity. Lecture Notes in Computer Science (including subseries Lecture Notes in Artificial Intelligence and Lecture Notes in Bioinformatics). 2018 4;11046 LNCS:55–63. 10.1007/978-3-030-00919-9_7 PMC6516490 31098597

[27] Krizhevsky A, Sutskever I, Hinton GE. ImageNet Classification with Deep Convolutional Neural Networks. In: Advances in Neural Information Processing Systems 25 (NeurIPS 2012). Curran Associates, Inc.; 2012. p. 1097–1105.

[28] He K, Zhang X, Ren S, Sun J. Deep residual learning for image recognition. In: Proceedings of the IEEE conference on computer vision and pattern recognition; 2016. p. 770–778.

[29] Ronneberger O, Fischer P, Brox T. U-Net: Convolutional Networks for Biomedical Image Segmentation. In: Navab N, Hornegger J, Wells WM, Frangi AF, editors. Medical Image Computing and Computer Assisted Intervention (MICCAI). vol. 9351. Cham: Springer International Publishing; 2015. p. 234–241. Available from: https://link.springer.com/chapter/10.1007/978-3-319-24574-4_28.

[30] Vaswani A, Shazeer N, Parmar N, Uszkoreit J, Jones L, Gomez AN, et al. Attention Is All You Need. In: Advances in Neural Information Processing Systems 30 (NeurIPS 2017). Curran Associates, Inc.; 2017. p. 6000–6010.

[31] Dosovitskiy A, Beyer L, Kolesnikov A, Weissenborn D, Zhai X, Unterthiner T, et al. An image is worth 16x16 words: Transformers for image recognition at scale. arXiv preprint arXiv:2010.11929. 2020;.

[32] Chen Z. Medical Image Segmentation Based on U-Net. In: Journal of Physics: Conference Series. vol. 2547. IOP Publishing; 2023. p. 12010.

[33] He K, Gan C, Li Z, Rekik I, Yin Z, Ji W, et al. Transformers in medical image analysis. Intell Med. 2023;3(1):59–78.

[34] MIM Software Inc .: MIM LiverY90. Available from: https://www.mimsoftware.com/sureplan-livery90/.

[35] Klein S, Staring M, Murphy K, Viergever MA, Pluim JPW. Elastix: a toolbox for intensity-based medical image registration. IEEE Trans Med Imaging. 2009;29(1):196–205. 19923044 10.1109/TMI.2009.2035616

[36] Isensee F, Jaeger PF, Kohl SAA, Petersen J, Maier-Hein KH. nnU-Net: a self-configuring method for deep learning-based biomedical image segmentation. Nat Methods. 2021;18(2):203–11. 33288961 10.1038/s41592-020-01008-z

[37] Quinton F, Popoff R, Presles B, Leclerc S, Meriaudeau F, Nodari G, et al. A tumour and liver automatic segmentation (ATLAS) dataset on contrast-enhanced magnetic resonance imaging for hepatocellular carcinoma. Data. 2023. 10.3390/data8050079.

[38] Janssens R, Zeng G, Zheng G. Fully automatic segmentation of lumbar vertebrae from CT images using cascaded 3D fully convolutional networks. In: IEEE 15th International Symposium on Biomedical Imaging (ISBI). vol. 2018-April; 2018. p. 893–897.

[39] Quinton F, Presles B, Leclerc S, Nodari G, Lopez O, Chevallier O, et al. Navigating the nuances: comparative analysis and hyperparameter optimisation of neural architectures on contrast-enhanced MRI for liver and liver tumour segmentation. Sci Rep. 2024;14(1):3522. 38347017 10.1038/s41598-024-53528-9 PMC10861452

[40] Kingma DP. Adam: A method for stochastic optimization. arXiv preprint arXiv:1412.6980. 2014.

[41] Taghanaki SA, Zheng Y, Zhou SK, Georgescu B, Sharma P, Xu D, et al. Combo loss: Handling input and output imbalance in multi-organ segmentation. Comput Med Imaging Graph. 2019;75:24–33. 31129477 10.1016/j.compmedimag.2019.04.005

[42] Taha AA, Hanbury A. Metrics for evaluating 3D medical image segmentation: analysis, selection, and tool. BMC Med Imaging. 2015;15:1–28. 26263899 10.1186/s12880-015-0068-x PMC4533825

[43] Pasciak AS, Bourgeois AC, Bradley YC. A comparison of techniques for 90Y PET/CT image-based dosimetry following radioembolization with resin microspheres. Front Oncol. 2014. 10.3389/fonc.2014.00121. 24904832 10.3389/fonc.2014.00121 PMC4033229

